# Supplementary material for: Exploring the Link Between Infections and Primary Osteoarthritis: A Next-Generation Metagenomic Sequencing Approach
Source: Int J Mol Sci. 2024 Dec 24;26(1):20. doi: 10.3390/ijms26010020 (PMC11720077; doi:10.3390/ijms26010020)
Supplement: Supplementary file 1 [file ijms-26-00020-s001.zip › ijms-3348776-supplementary.docx]

**Exploring the link between infections and primary osteoarthritis: a next-generation metagenomic sequencing approach**

**Supplementary Materials**

**Material and Methods**

**Table S1.** The patients’ medical history, preoperative laboratory test results, and course of treatment

| **Patients’ medical history** | **Patient A** | **Patient B** |
| --- | --- | --- |
| **Alloplasty** | | |
| Implant fixation | Cementless | Cemented |
| Implant type | Triathlon PS Stryker | Triathlon TS Stryker |
| Femoral implant size | 5 | 5 |
| Tibial implant size | 4 | 6 |
| Insert size | 9 mm | 11 mm |
| Tibial/femoral stem | None | Tibial |
| Anesthesia | General | Spinal |
| Surgery duration | 100 min | 80 min |
| **Orthopedic Examination (Pre-Op)** | | |
| Lateralization | Left | Left |
| Kellgren–Lawrence grade | 3/4 | 4 |
| Lower limb axis | Varus | Varus |
| Intra-articular injections | None | None |
| Range of motion (0°–150°) | 10°–130° | 0°–100° |
| Number of symptomatic years | 46 years (exacerbation for 1 year) | 4 years |
| **Knee type classification** | I | VII |
| Anatomical Medial Proximal Femoral Angle (aMPFA) (80–89º) | 98.8° | 93.7° |
| Mechanical Lateral Proximal Femoral Angle (mLPFA) (85–90º) | 81.2° | 86.3° |
| Medial Proximal Tibial Angle (MPTA) (85–90º) | 84.4° | 79.3° |
| Anatomical Lateral Distal Tibial Angle (aLDTA) (86–92º) | 95.6° | 100.7° |
| Posterior slope | 6.1° | 8.6° |
| Mechanical Axis Deviation (MAD) | 32 mm | 82 mm |
| **Blood results** | | |
| Leukocytes (WBC) (3.9–10.0 ×10^3/μl) | 7.3 10^3/μl | 6.7 10^3/μl |
| Erythrocytes (RBC) (3.90–5.60 ×10^6/μl) | 4.74 10^6/μl | 4.83 10^6/μl |
| Hemoglobin (HGB) (11.20–16.47 g/dl) | 14.20 g/dl | 14.70 g/dl |
| Hematocrit (HCT) (34.1–51.0%) | 42.5 % | 42.8 % |
| Mean corpuscular volume (MCV) (79.0–94.8 fl) | 89.7 fl | 88.6 fl |
| Mean corpuscular hemoglobin (MCH) (25.6–32.2 pg) | 30.0 pg | 30.4 pg |
| Mean corpuscular hemoglobin concentration (MCHC) (32.2– 36.5 g/dl) | 33.4 g/dl | 34.3 g/dl |
| Red Cell Distribution Width (RDW) (11.6–14.4%) | 12.6 % | 13.8 % |
| Red Cell Distribution Width (RDW) (35.1–46.3 fl) | 41.3 fl | 44.5 fl |
| Platelets (PLT) (125–396 10^3/μl) | 312 10^3/μl | 286 10^3/μl |
| Plateletcrit (PCT) (0.16–0.38%) | 0.33 % | 0.33 % |
| Platelet Distribution Width (PDW) (9.8–16.2 fl) | 12.4 fl | 14.1 fl |
| Mean Platelet Volume (MPV) | 10.6 fl | 11.4 fl |
| Platelet-large cell ratio (P-LCR) (19.2–47.0%) | 30.0 % | 35.6 % |
| Neutrophils (NEU%) (40.80–70.39%) | 51.90 % | 57.40 % |
| Lymphocytes (LYMPH%) (21.0 - 50.0%) | 36.9 % | 21.5 % |
| Monocytes (MON%) (5.1–11.2%) | 8.9 % | 11.9 % |
| Eosinophils (EOS%) (0.4–6.6%) | 1.8 % | 8.4 % |
| Basophils (BASO%) (0.2–1.3%) | 0.5 % | 0.8 % |
| Immature granulocytes % (0.0–0.5%) | 0.3 % | 0.3 % |
| Neutrophils (NEU) (1.78–6.04 ×10^3/μl) | 3.81 10^3/μl | 3.82 10^3/μl |
| Lymphocytes (LYMPH) (1.3– 3.4 ×10^3/μl) | 2.7 10^3/μl | 1.4 10^3/μl |
| Monocytes (MON) (0.31–0.92 ×10^3/μl) | 0.65 10^3/μl | 0.79 10^3/μl |
| Eosinophils (EOS) (0.03– 0.39 ×10^3/μl) | 0.13 10^3/μl | 0.56 10^3/μl |
| Basophils (BASO) (0.01–0.09 ×10^3/μl) | 0.04 10^3/μl | 0.05 10^3/μl |
| Immature granulocytes (< 0.04 ×10^3/μl) | 0.02 10^3/μl | 0.02 10^3/μl |
| Erythrocyte sedimentation rate (ESR) (< 20 mm/h) | 2 mm/h | 2 mm/h |
| Sodium (136–145 mmol/L) | 143 mmol/l | 139 mmol/l |
| Potassium (3.5–5.1 mmol/L) | 4.8 mmol/l | 4.7 mmol/l |
| Creatinine (0.5–1.2 mg/dl) | 0.7 mg/dl | 1.0 mg/dl |
| eGFR | 88.5 ml/min/1.73m^2 | 77.5 ml/min/1.73m^2 |
| Urea (15–40 mg/dl) | 36 mg/dl | 38 mg/dl |
| Glucose (70–99 mg/dl) | 91 mg/dl | 98 mg/dl |
| **Urinalysis** | | |
| Color | light yellow | yellow |
| Transparency | transparent | transparent |
| specific gravity (1.005–1.030) | 1,012 | 1,024 |
| pH (5.0–7.5) | 5 | 6 |
| Protein (absent) | absent | 300.0 mg/dl |
| Glucose (absent) | absent | absent |
| Urobilinogen (normal) | normal | normal |
| Bilirubin (absent) | absent | absent |
| Ketone bodies (absent) | absent | absent |
| Leukocytes (absent) | absent | absent |
| Nitrites (absent) | absent | absent |
| Erythrocytes (absent) | present | present |
| **Microscopic Urine Sediment** | | |
| Squamous epithelial cells (0–2 /hpf) | 1 /hpf | 1 /hpf |
| Renal epithelial cells (0–1 /hpf) | 0–1 /hpf | 0 /hpf |
| Leukocytes (0–2 /hpf) | 0–8 /hpf | 3–5 /hpf |
| Erythrocytes (0–2 /hpf) | 0 – 3 /hpf | 5–10 /hpf |
| Bacteria (absent) | individual /hpf | numerous /hpf |
| Single strands of mucus | numerous /hpf | numerous /hpf |

hpf; high power field

**Microbiological examination of biological samples: microorganism culture**

Approximately 0.1 mL of sample was taken from each of the collected joint samples and placed in two tubes containing 5 ml of Brain Heart Infusion (BHI) broth: BHI NAD and BHI with hemin and vitamin K (Becton, Dickinson and Company, USA) to determine the presence of culturable microorganisms. Subsequently, the tubes were dispatched to the Department of Medical Microbiology at the Medical University of Warsaw for microbiological examination *via* classical culture methods. No microbial growth was observed in any of the cultures following a seven-day incubation period.

**DNA isolation result**

**Table S2.** The concentration, mass, and quality of DNA obtained during sample isolation

| **Sample** | **Patient** | **Sample code** | **Concentration (ng/μl)** | **Volume (μl)** | **Total mass(μg)** | **Q test result** | **Remark** |
| --- | --- | --- | --- | --- | --- | --- | --- |
| **Blood** | A | Ab1 | 27.5 | 40 | 1.1 | Level A | N/A |
|  |  | Ab2 | 35.5 | 40 | 1.42 | Level A | N/A |
|  | B | Bb1 | 66.5 | 40 | 2.66 | Level A | N/A |
|  |  | Bb2 | 90.6 | 40 | 3.624 | Level A | N/A |
| **Synovial fluid** | A | Af1 | 4.5 | 40 | 0.18 | Level C | c<12.5ng/μl. m<0.5μg |
|  |  | Af2 | 4.5 | 40 | 0.18 | Level C | c<12.5ng/μl. m<0.5μg |
|  | B | Bf1 | 3.5 | 40 | 0.14 | Level C | c<12.5ng/μl. m<0.5μg |
|  |  | Bf2 | 3.6 | 40 | 0.15 | Level C | c<12.5ng/μl. m<0.5μg |
| **Synovial tissue** | A | At1 | 110.4 | 40 | 4.416 | Level A | N/A |
|  |  | At2 | 76.0 | 40 | 3.04 | Level A | N/A |
|  | B | Bt1 | 179.7 | 40 | 7.188 | Level A | N/A |
|  |  | Bt2 | 510.4 | 40 | 20.416 | Level A | N/A |

Key

Level A: the sample meets the requirements of library construction and sequencing. The current success rate is around 93.59%

Level C: the sample does not meet the requirements of library construction and sequencing. The current success rate is around 70.53%

The quality of all joint fluid samples from both patients did not meet the required standards for successful library construction. The probability of success was estimated to be 70%. However, given the diagnostic value of the samples and the inability to re-sample, we deemed it prudent to accept the risk of non-success and utilize them for subsequent stages of the study. Subsequently, library construction was successfully completed, with all samples meeting the required criteria for sequencing.

**Library construction**

The library construction process involved several steps. First, the target sequences were fragmented and sized to a desired length. Then, the target was converted into double-stranded DNA, and oligonucleotide adapters were attached to the ends of the target fragments. Ultimately, the final library product was quantified for sequencing.

For sample disruption, 1 μg of genomic DNA was disrupted using a Covaris ultrasonicator (Covaris, LLC., USA).

For fragment size selection, after disruption, DNA fragments in the range of 200–400 bp were concentrated by fragment size selection using magnetic beads (MGI Tech Co., China).

In the next step, the DNA fragments underwent end-repair. A single adenine base was added to the 3′end *via* an A-tailing reaction. This A-overhang allows adapters containing a single thymine over-hanging base to base pair with the DNA fragments. Polymerase chain reaction (PCR) was used for amplification of specific DNA fragments. The amplified products were purified and recovered using magnetic beads.

For cyclization of the product, the double-stranded DNA (dsDNA) was denatured into single strands (ssDNA) using the MGIEasy Universal Library Conversion Kit. This conversion process involved transforming linear dsDNA libraries into single-stranded circular DNA (ssCirDNA) libraries. Following digestion of the linear uncyclized DNA molecule, the final library was obtained.

**Library Quality Control**: The concentration of the cyclization product was determined using a Qubit 4 fluorometer (Invitrogen). The ExKubit dsDNA Assay Kit and Agilent 2100 (Agilent Technologies, CA) were employed for fragment length control. For construction of the library, double-distilled water was used as a negative control, and no DNA content (0 ng/ml) was detected during the procedure.

**Data Filtering**

A summary of the raw data and quality control (QC) results is shown in the Table S3. The sequencing pathway yielded a total of over three hundred and fifty million reads for each sample. Following removal of low-quality sequences, adapter sequences, and sequences potentially originating from the host, more than one hundred and thirty million base pairs of clean data were obtained for each sample. The proportion of non-host DNA ranged from 0.3% to 0.8%, with the level varying according to the sample. A lower level of 0.3% was detected in all joint tissue samples from both patients. In the remaining samples, the mean level of non-host-derived DNA content was approximately 0.5%.

**Table S3.** Data filtering raw data and quality control result.

| Sample Name | Patient | Sample code | Raw reads count | Raw bases count | Clean reads  count after QC | Clean bases count after QC | Clean data  rate (%) | Q20 base content (%) | Q30 base content (%) | GC content (%) | Clean  reads  after host removal | Clean  bases  after host removal | Host rate  % |
| --- | --- | --- | --- | --- | --- | --- | --- | --- | --- | --- | --- | --- | --- |
| Blood | A | Ab1 | 352160000 | 52824000000 | 333500730 | 50025109500 | 94.7 | 97.69 | 92.57 | 41.17 | 1767970 | 265195500 | 99.469875 |
|  |  | Ab2 | 351760000 | 52764000000 | 333502702 | 50025405300 | 94.81 | 95.47 | 88.52 | 40.83 | 1801512 | 270226800 | 99.459821 |
|  | B | Bb1 | 348960000 | 52344000000 | 333564272 | 50034640800 | 95.59 | 98.35 | 93.84 | 41.47 | 1393754 | 209063100 | 99.582163 |
|  |  | Bb2 | 351040000 | 52656000000 | 333535156 | 50030273400 | 95.01 | 98.4 | 94.21 | 41.66 | 1541344 | 231201600 | 99.537877 |
| Synovial fluid | A | Af1 | 354320000 | 53148000000 | 333538904 | 50030835600 | 94.13 | 98.0 | 93.46 | 41.34 | 1366722 | 205008300 | 99.590236 |
|  |  | Af2 | 361200000 | 54180000000 | 333502696 | 50025404400 | 92.33 | 98.41 | 93.91 | 41.37 | 2835812 | 425371800 | 99.149688 |
|  | B | Bf1 | 354560000 | 53184000000 | 333669106 | 50050365900 | 94.11 | 97.81 | 92.7 | 41.65 | 1512470 | 226870500 | 99.546716 |
|  |  | Bf2 | 348400000 | 52260000000 | 333640134 | 50046020100 | 95.76 | 98.07 | 94.08 | 40.82 | 1312582 | 196887300 | 99.606587 |
| Synovial tissue | A | At1 | 346960000 | 52044000000 | 334197366 | 50129604900 | 96.32 | 97.54 | 92.34 | 40.67 | 1013114 | 151967100 | 99.696852 |
|  |  | At2 | 346960000 | 52044000000 | 334054872 | 50108230800 | 96.28 | 97.64 | 92.57 | 40.57 | 911356 | 136703400 | 99.727184 |
|  | B | Bt1 | 340480000 | 51072000000 | 333615846 | 50042376900 | 97.98 | 98.01 | 93.09 | 41.45 | 904066 | 135609900 | 99.72901 |
|  |  | Bt2 | 339200000 | 50880000000 | 333764858 | 50064728700 | 98.4 | 98.0 | 93.26 | 40.52 | 916420 | 137463000 | 99.725429 |

**Assembly Results**

Following quality control, the shorter fragments were filtered out. The resulting clean data underwent assembly, statistical analysis, and subsequent gene prediction using MEGAHIT software (version 1.2.9). For the purpose of gene prediction, the assembled data was subjected to comparative analysis with sequences of known microorganisms in the database to predict coding genes, specifically protein-coding sequences (CDSs). In the next step, a high-quality gene prediction catalog was obtained through a de-redundancy operation. The assembly results and gene prediction result are shown in Table S4.

**Table S4.** Assembly and gene prediction results

| Sample Name | Patient | Sample code | Contig number | Assembly length | N50 | N90 | Max | Min | Average size | Predicted  CDS number |  | CDS number  after duplicates  removed |
| --- | --- | --- | --- | --- | --- | --- | --- | --- | --- | --- | --- | --- |
| Blood | A | Ab1 | 18338 | 9935773 | 515 | 323 | 16282 | 300 | 541 | 37500 |  | 19782 |
|  |  | Ab2 | 14507 | 7954297 | 529 | 327 | 9536 | 300 | 548 | 29006 |  | 16614 |
|  | B | Bb1 | 10933 | 6708273 | 648 | 340 | 10327 | 300 | 613 | 20837 |  | 13386 |
|  |  | Bb2 | 11678 | 7119916 | 639 | 338 | 13263 | 300 | 609 | 22636 |  | 14315 |
| Synovial fluid | A | Af1 | 12046 | 7120744 | 609 | 334 | 9700 | 300 | 591 | 24054 |  | 14492 |
|  |  | Af2 | 14371 | 8524436 | 610 | 339 | 18800 | 300 | 593 | 29773 |  | 17817 |
|  | B | Bf1 | 9742 | 6132851 | 670 | 351 | 11809 | 300 | 629 | 18961 |  | 12952 |
|  |  | Bf2 | 8805 | 5365841 | 644 | 340 | 10837 | 300 | 609 | 16417 |  | 10904 |
| Synovial tissue | A | At1 | 10725 | 6178811 | 585 | 331 | 9431 | 300 | 576 | 20975 |  | 12802 |
|  |  | At2 | 9690 | 5661821 | 594 | 332 | 8146 | 300 | 584 | 18756 |  | 11710 |
|  | B | Bt1 | 7139 | 4518890 | 675 | 348 | 7302 | 300 | 632 | 11742 |  | 8233 |
|  |  | Bt2 | 7232 | 4528519 | 668 | 347 | 7584 | 300 | 626 | 12350 |  | 8548 |

Key: Following quality control, the shorter fragments were filtered out. The resulting clean data underwent assembly, statistical analysis, and subsequent gene prediction using MEGAHIT software (version 1.2.9). For the purpose of gene prediction, the assembled data was subjected to comparative analysis with sequences of known microorganisms in the database to predict coding genes, specifically protein-coding sequences (CDSs). In the next step, a high-quality gene prediction catalog was obtained through a de-redundancy operation.

The results of the assembly were evaluated in terms of continuity (assembly length, max length) and integrity (N50, N90, min length). N50 represents the sorting and accumulation of contig/scaffold lengths from the longest to the shortest. Once the cumulative sum reaches 50% of the total length of the contig/scaffold, the length of the last contig/scaffold is defined as the contig/scaffold N50. Similarly, the N90 is defined.

**Taxonomic distribution**
**Figure S1.** Patient A. Krona plots; each ring from the inside to the outside represents the different taxon levels of kingdom, phylum, class, order, family, genus and species and the abundance of the taxa. Double-click on the arc segment of a high level to view the taxa at that level.

<https://eu-biosys.bgi.com/project/production/BGI_659f4de6b3c87a6a396d4a57/TaxonomyAnalysis/Distribution/Krona/AbAfAt/krona_table.html?dataset=0&node=0&collapse=true&color=false&depth=7&font=11&key=true>

**Figure S2.** Patient B. Krona plots; each ring from the inside to the outside represents the different taxon levels of kingdom, phylum, class, order, family, genus and species and the abundance of the taxa. Double-click on the arc segment of a high level to view the taxa at that level.

<https://eu-biosys.bgi.com/project/production/BGI_659f4de6b3c87a6a396d4a57/TaxonomyAnalysis/Distribution/Krona/BbBfBt/krona_table.html?dataset=0&node=0&collapse=true&color=false&depth=7&font=11&key=true>

**Gene diversity**

Gene alpha diversity 
The diversity index describes the diversity of microbial communities within a biological sample (Figure S3) and group of samples from a patient (Figure S4). To determine how the diversity differs between samples non-parametric tests were used. 
No diversity was found within a particular biological sample. In samples taken from both patients, diversity in gene abundance was found.

**Figure S3.** Gene alpha diversity box plot.


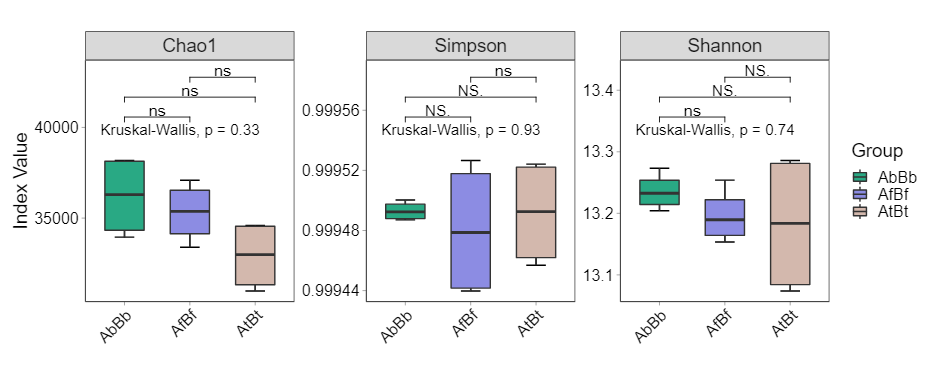


Key: The abscissa and different boxes indicate groups of samples (b - blood, f - fluid, t - tissue) taken from patients (A, B).

The ordinates indicate the index values. Horizontal line above the column connects two analyzed groups; p values between two groups were calculated, p <0.05 indicates a significant difference of alpha indices between the groups of biological samples, ns indicates p >0.05, and NS indicates p =1.

**Figure S4**. Gene alpha diversity box plot.


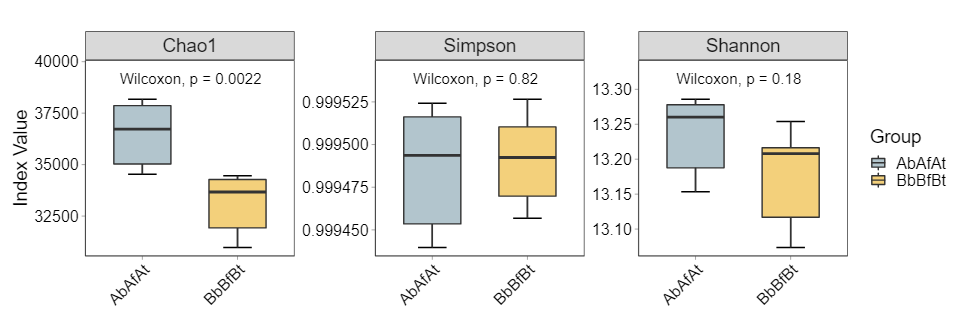


Key: Each box plot represents a diversity index. The abscissa and different boxes indicate groups of samples (b - blood, f - fluid, t - tissue) taken from individual patients (A, B); the ordinates indicate the index values; *p* <0.05 indicates a significant difference in alpha indices between the patients.

**Gene beta diversity**

To analyze beta diversity, the gene abundance information for each sample was used to calculate the distance or similarity between samples. The analysis revealed that no significant differences in microbial communities among biological samples (Figure S5); however, a significant difference in microbial communities was found between patients (p=0.000056) (Figure S6).

**Figure S5.** Gene beta diversity box plot (Bray–Curtis calculation)


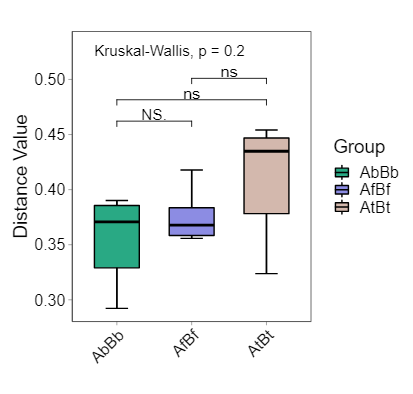


Key: The color of the abscissa and box indicates different groups; the ordinate indicates the distance between samples; the upper and lower edges of the box represent the first quartile and lower quartile, respectively, of distances within the group. The horizontal line within the box represents the median of distances, and the ends of the straight line above and below the box represent the maximum and minimum distances, respectively. Each box reflects the distance distribution within the samples. The greater the median, the greater the sample distances within the group; the longer the box and the straight line outside the box, the greater the distance differences within the group. A horizontal line above the column connects groups samples, ns indicates p >0.05, and NS indicates p =1. 

**Figure S6**. Gene beta diversity box plot (the Bray–Curtis calculation)


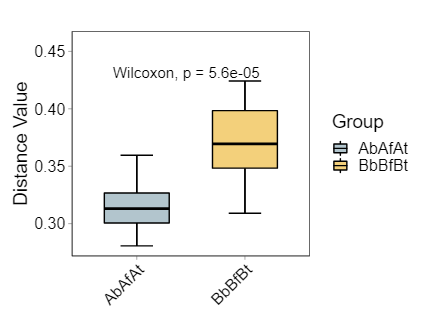


Key: The color of the abscissa and box indicates different patients; the ordinate indicates the distance between patients. The upper and lower edges of the box represent the first quartile and lower quartile, respectively, of distances within the group. The horizontal line within the box represents the median of distances. The ends of the straight line above and below the box represent the maximum corresponding group. The greater the median, the greater the sample distances within the group; the longer the box and the straight line outside the box, the greater the distance differences within the group; p <0.05 indicates a significant difference.
